# Delivering CRISPR: a review of the challenges and approaches

**DOI:** 10.1080/10717544.2018.1474964

**Published:** 2018-05-25

**Authors:** Christopher A. Lino, Jason C. Harper, James P. Carney, Jerilyn A. Timlin

**Affiliations:** Bioenergy and Defense Technologies, Sandia National Laboratories, Albuquerque, NM, USA

**Keywords:** CRISPR, Cas9, history, overview, review, drug delivery, prospective

## Abstract

Gene therapy has long held promise to correct a variety of human diseases and defects. Discovery of the Clustered Regularly-Interspaced Short Palindromic Repeats (CRISPR), the mechanism of the CRISPR-based prokaryotic adaptive immune system (CRISPR-associated system, Cas), and its repurposing into a potent gene editing tool has revolutionized the field of molecular biology and generated excitement for new and improved gene therapies. Additionally, the simplicity and flexibility of the CRISPR/Cas9 site-specific nuclease system has led to its widespread use in many biological research areas including development of model cell lines, discovering mechanisms of disease, identifying disease targets, development of transgene animals and plants, and transcriptional modulation. In this review, we present the brief history and basic mechanisms of the CRISPR/Cas9 system and its predecessors (ZFNs and TALENs), lessons learned from past human gene therapy efforts, and recent modifications of CRISPR/Cas9 to provide functions beyond gene editing. We introduce several factors that influence CRISPR/Cas9 efficacy which must be addressed before effective *in vivo* human gene therapy can be realized. The focus then turns to the most difficult barrier to potential *in vivo* use of CRISPR/Cas9, delivery. We detail the various cargos and delivery vehicles reported for CRISPR/Cas9, including physical delivery methods (e.g. microinjection; electroporation), viral delivery methods (e.g. adeno-associated virus (AAV); full-sized adenovirus and lentivirus), and non-viral delivery methods (e.g. liposomes; polyplexes; gold particles), and discuss their relative merits. We also examine several technologies that, while not currently reported for CRISPR/Cas9 delivery, appear to have promise in this field. The therapeutic potential of CRISPR/Cas9 is vast and will only increase as the technology and its delivery improves.

## Introduction

Discovery of the Clustered Regularly-Interspaced Short Palindromic Repeats (CRISPR) (Ishino et al., 1987; Mojica et al., 1993; van Soolingen et al., 1993), their function as part of an adaptive prokaryotic immune system (CRISPR-associated system, Cas) (Bolotin et al., 2005; Mojica et al., 2005; Pourcel et al., 2005; van der Oost et al., 2009), and subsequent development into a genomic editing tool (Jinek et al., 2012; Cho et al., 2013; Cong et al., 2013; Mali et al., 2013), has revolutionized the field of molecular biology. Much of this enthusiasm centers on the clinical potential of CRISPR/Cas9 for treating human disease and editing the human genome. However, the simplicity and specificity with which CRISPR/Cas9 can edit DNA is changing the pace of biological research in many areas, including identifying and understanding mechanisms of genetic diseases (Findlay et al., 2014; Gilbert et al., 2014; Zhou et al., 2014; Konermann et al., 2015), validating disease targets (Shalem et al., 2014; Wang et al., 2014), developing animal disease models (Wang et al., 2013; Yang et al., 2013), facilitating genetic engineering in plants (Raitskin & Patron, 2016; Zhang et al., 2016, 2017), and allowing for more thorough epigenetic studies (Yao et al., 2015; Vora et al., 2016). This broad impact of the CRISPR/Cas9 gene editing tool has led to over 6000 research publications since its development five years ago.

Gene therapy may greatly benefit from CRISPR/Cas9 technology. To date, over 3000 genes have been associated with disease-causing mutations (Cox et al., 2015). Early efforts to correct disease-causing genetic mutations in humans, although generally successful, were tainted by several tragedies. Perhaps the most well-known early gene therapy trial involved two studies from France (Hacein-Bey-Abina et al., 2002; Hacein-Bey-Abina et al., 2010) and the UK (Gaspar et al., 2004; Gaspar et al., 2011) of children suffering from X-linked severe combined immunodeficiency (SCID X-1). Of the 20 patents participating in the trial, 17 were successfully and stably cured (Cavazzana et al., 2016). However, five children subsequently developed T-cell leukemia, with one child dying from chemotherapy-refractory leukemia. In all cases of leukemia, the SCID X-1 correcting gene had inserted into the patient genome within or near tumor-promoting genes and caused transcriptional activation (Check, 2002; Kaiser, 2003; Thomas et al., 2003). In another tragedy, an 18-year-old male suffering from a partial deficiency of ornithine transcarbamylase (OTC) died after developing a massive inflammatory response to the genetic cargo delivery vehicle, an adenovirus vector, four hours after receiving the treatment (Marshall, 1999; Authors, 2002).

It is important to note that both tragedies stemmed from the therapeutic delivery method (LaFountaine et al., 2015). In the case of SCID X-1, the correcting gene construct was non-specifically inserted into the genome; in the case of partial OTC, the viral vector induced a severe immune response. It is therefore critical that gene therapy technologies allow for highly specific editing of the genome to reduce the risk of undesired mutagenesis, and that the delivery vehicle allows for safe and efficient transport to the target.

In just one decade after these tragedies, great progress has been made in advancing gene therapy technologies, leading to renewed enthusiasm in the promise of broad-spectrum treatment of genetic diseases. Advances include the discovery and development of site-specific nucleases for gene editing: zinc finger nucleases (ZFNs) (Bibikova et al., 2002), transcription activator-like effector nucleases (TALENs) (Christian et al., 2010), and CRISPR/Cas9. Advances further include tools for delivery of the cargo to targeted cells for genetic editing, both *ex vivo* and *in vivo*. Careful consideration and development of both the gene editing tool and the delivery mechanism will be required if the full potential of therapeutic gene editing is to be realized. This review will briefly introduce ZFNs and TALENs, then provide an in-depth description of the CRISPR/Cas9 system, including recent advances and modifications to the technology, and factors affecting system performance. This will be followed by a comprehensive synopsis of existing CRISPR/Cas9 delivery methods, their potential and challenges in delivering CRISPR, and recently reported promising candidates for delivery of gene editing systems.

## Gene editing

**Figure 1.**
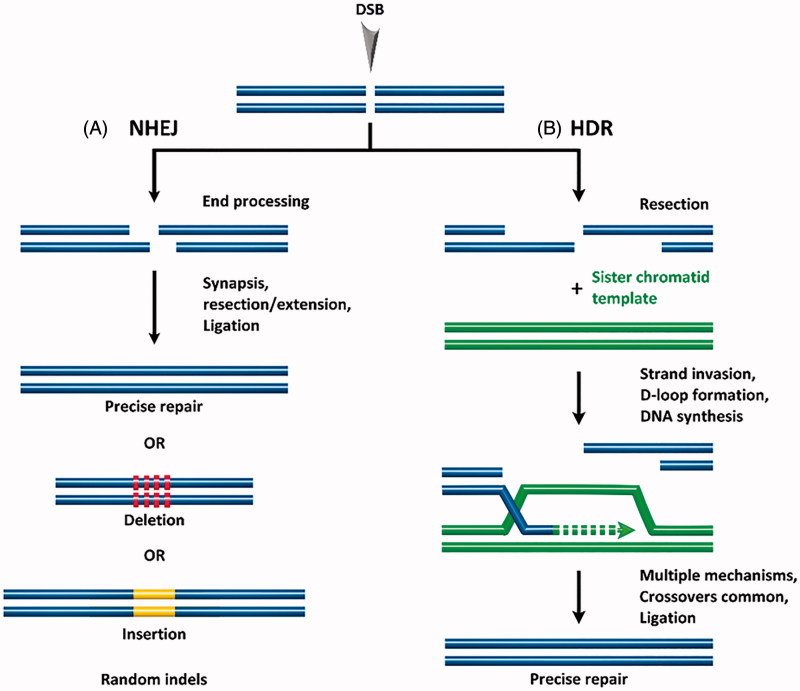
Following formation of a double stranded break (DSB), endogenous DNA repair can occur by (A) non-homologous end joining (NHEJ) resulting in random indels, or by (B) homology-directed repair (HDR) which uses a template DNA strand for precise repair.

At the core, gene gain/loss-of-function therapy comprises (1) the generation of double-stranded breaks (DSBs) in defined regions of the genome, (2) correction of the defective endogenous genes or introduction of exogenous genes, and (3) DSB repair. DSBs in eukaryotes are repaired by one of two endogenous repair mechanisms: non-homologous end joining (NHEJ) or homology-directed repair (HDR). In NHEJ, protein factors re-ligate the broken DNA strand either directly or by including nucleotide insertions or deletions (indels) (Hefferin & Tomkinson, 2005). This process occurs without a homologous DNA template, regularly leading to mutations and deletions in the repaired strand (Bibikova et al., 2002), as shown in Figure 1(A). NHEJ is, therefore, characterized as error-prone. NHEJ can occur at any phase of the cell cycle and is the primary cellular DSB repair mechanism. In contrast, HDR uses a homologous repair template to precisely repair the DSB (Capecchi, 1989; Takata et al., 1998) (see Figure 1(B)). HDR typically occurs in late S- or G_2_-phase, when a sister chromatid can serve as the repair template. In general, the incidence of HDR for DSB repair is extremely low compared with NHEJ, at least in instances where both pathways are equally available to an organism. Given the significant gene editing enabled by HDR, development of methods to increase the incidence/efficiency of HDR for gene editing with site-specific nucleases is an active field of research.

**Figure 2.**
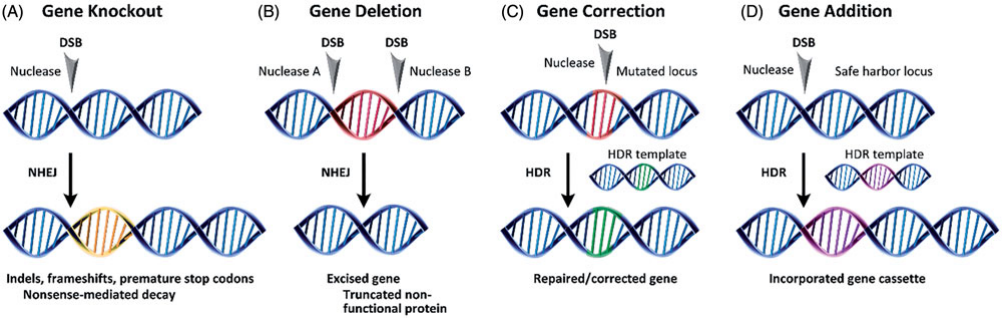
Products of site-specific nuclease-based gene editing: (A) gene knockout, (B) gene deletion, (C) gene correction, and (D) gene addition.

The use of site-specific nucleases and NHEJ or HDR generally results in one of four gene editing products. Shown in Figure 2, these include gene knockout, deletion, correction, or addition. The error-prone character of NHEJ can be exploited to introduce indels and frameshifts into the coding regions of a gene. This knocks the gene out (Figure 2(A)) via nonsense-mediated decay of the mRNA transcript. In gene deletion (Figure 2(B)), paired nucleases excise regions of the coding gene, resulting in premature truncation and knockout of the protein in a manner more generally efficient than introducing frameshifts. Both gene correction (Figure 2(C)) and gene addition (Figure 2(D)) require an exogenous DNA template that can be introduced as either single-stranded (Radecke et al., 2010; Chen et al., 2011; Soldner et al., 2011) or double-stranded DNA (Rouet et al., 1994). The DNA template contains homologous sequence arms that flank the region containing the desired mutation or gene cassette.

## Site-specific nucleases

To address challenges with non-specific insertion, provide greater fidelity, and assist in more precise gene editing, programable nucleases have been developed.

### Zinc finger nucleases (ZFNs)

**Figure 3.**
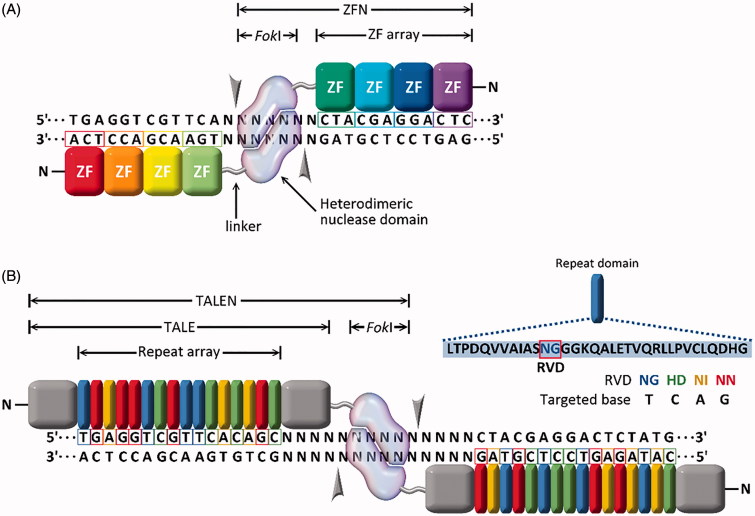
Site-specific endonucleases with programable DNA-binding protein domains: (A) zinc finger nucleases (ZFNs) and (B) transcription activator-like effector nucleases (TALENs).

In 2002, the first sequence-specific nucleases, termed zinc finger nucleases (ZFNs), were reported by Bibikova et al. (2002, 2003). ZFNs are a fusion protein of Cys2-His2 zinc finger proteins (ZFPs) and a non-specific DNA restriction enzyme derived from *Fok*I endonucleases, as shown in Figure 3(A). ZFPs are common in eukaryotic cells and are associated with transcriptional regulation and protein–protein interactions (Wolfe et al., 2000; Urnov et al., 2010). For an in-depth review on specific ZFN function, see the review by Carroll (2011).

Challenges with ZFNs include design and engineering of the ZFP for high-affinity binding of the desired sequence, which can prove non-trivial (Ramirez et al., 2008). Also, not all sequences are available for ZFP binding, so site selection is limited. Using open-source ZFP domains, sites could be targeted only every 200-bps in a random DNA sequence (Gupta & Musunuru, 2014). This may not be a concern if the objective is gene knockout or deletion; however, this may be an obstacle if the objective is a gene correction or addition product. Another significant challenge is off-target cutting (Gabriel et al., 2011; Pattanayak et al., 2011). ZFN design improvements addressing off-target concerns have included ZFNs that work in pairs, with each pair recognizing two sequences that flank the target cleavage site. One ZFN binds the forward strand, and the second ZFN binds the reverse strand, increasing the total number of recognized bps to between 18 and 36. Further, *Fok*I domains that are obligate heterodimers with opposite charge have been fused to ZFPs such that only properly paired ZFNs will result in *Fok*I dimerization/activity and the formation of a DSB (see Figure 3(A)) (Miller et al., 2007; Doyon et al., 2011).

### Transcription activator-like effector nucleases (TALENS)

Four years following the development of ZFNs, a new class of natural DNA-binding proteins was discovered in the plant pathogenic bacteria *Xanthomonas sp.* (Zhang et al., 2013). The proteins, termed transcription activator-like effectors (TALEs), contain 33–35 amino acid repeats that flank a central DNA binding region (amino acids 12 and 13). This DNA binding region, known as the repeat variable di-residues (RVDs), specifically binds the DNA (Christian et al., 2010; Miller et al., 2011) as shown in Figure 3(B). Shortly after the discovery of TALEs, TALE nucleases (TALENs) were developed that, like ZFNs, are a fusion protein comprised of a TALE and a *Fok*I nuclease (Christian et al., 2010; Miller et al., 2011; Li et al., 2011; Reyon et al., 2012). For an in-depth TALEN function review, see Joung & Sander (2013).

Unlike ZFNs, design and engineering of TALENs is much simpler and can be done in a shorter time (Cermak et al., 2011; Reyon et al., 2012). TALENs are also not as limited as ZFNs in target site selection due to the 1:1 TALE–DNA binding (Zhu et al., 2013). While off-target cutting remains a concern, TALENs have been shown in one side-by-side comparison study to be more specific and less cytotoxic than ZFNs (Mussolino et al., 2014). TALENs, however, are substantially larger than ZFNs, requiring 3 kb of cDNA encoding for one TALEN versus just 1 kb for a single ZFN. This makes delivery of a pair of TALENs more challenging than a pair of ZFNs due to delivery vehicle cargo size limitations. Further, packaging and delivery of TALENs in some viral vectors may be problematic due to the high level of repetition in the TALENs sequence.

### CRISPR/Cas9

The most recently developed site-specific gene editing tool, CRISPR/Cas9, is a naturally occurring RNA-guided endonuclease. A methodical investigation by the scientific community has deciphered the natural function of the CRISPR/Cas9 gene editing system. Based on this work, several laboratories developed CRISPR/Cas9 as a tool that has now been applied in much of modern molecular biology. A key difference of this system from the protein-based binding to DNA of ZFNs and TALENs is the use of a short RNA sequence as the specificity-determining element to drive the formation of a DSB at the targeted site. The use of CRISPR/Cas9 avoids the need for protein engineering to develop a site-specific nuclease against a specific DNA target sequence, requiring only the synthesis of a new piece of RNA. This dramatically simplifies and greatly reduces the time needed for gene editing design and implementation.

## History of CRISPR

Discovery of unusual repeat sequences in *E. coli* separated by non-repeating sequences in a nearly palindromic pattern was first reported by Ishino et al. (1987). Described as ‘curious sequences’, similar sequences were identified in *Haloferax* and *Haloarcula* archaea by Mojica et al. (1993) and in *M. tuberculosis* by van Soolingen et al. (1993). The function of the interrupted repeat sequences was unknown, but they were soon identified in 20 microbial species (Mojica et al., 2000) and later found in more than 40% of bacteria and 90% of archaea (Mojica et al., 2005). In 2002, the acronym CRISPR was proposed to bring uniformity to the description of the sequences.

Two significant advances in the understanding of the CRISPR system were then made in 2002, when Jansen discovered a set of genes adjacent to the CRISPR locus, which was termed CRISPR-associated system, or Cas (Jansen et al., 2002). Analysis of the genes indicated a functional relationship between the CRISPR/Cas genes/loci and involvement in DNA metabolism or gene expression. However, the function remained a mystery. The second significant advance occurred in 2005 when Mojica et al. (2005), Pourcel et al. (2005), and Bolotin et al. (2005) all independently reported that the non-repeating CRISPR spacers contained sequences derived from foreign chromosomal DNA, specifically DNA from bacteriophages. Further, some bacteria that carried a given viral DNA sequence in the CRISPR locus were known to be resistant to infection by that phage, indicating that the CRISPR system may be a type of adaptive immune system in prokaryotes. All three studies hypothesized an adaptive immune system function of CRISPR and were rejected by high-profile journals, eventually being published elsewhere (Lander, 2016). The first experimental evidence of this hypothesis was published by Barrangou et al. (2007).

## Biological mechanism of CRISPR

Following discovery of the native CRISPR system function in bacteria, researchers set out to understand the mechanism of the adaptive immune system. Although initially hypothesized to follow a RNA interference mechanism (Mojica et al., 2005), it was quickly determined that CRISPR functions as a genomic memory of invading pathogens. This memory is used by Cas proteins, serving as guided endonucleases, to scan for invading DNA and disable it by introducing DSBs (Brouns et al., 2008).

CRISPR systems were further classified into six types that were additionally grouped into two classes (Wiedenheft et al., 2011; Wright et al., 2016). Types I–III are the most studied, while types IV–VI were more recently identified (Makarova & Koonin, 2015; Makarova et al., 2015; Shmakov et al., 2015). Type I and Type III CRISPR systems both utilize sets of Cas proteins. In Type I systems, a multi-protein CRISPR RNA (crRNA) complex known as Cascade recognizes the target DNA, which is then cleaved by Cas3. In Type III systems, Cas10 assembles into a Cascade-like complex that recognizes and cleaves the target.

**Figure 4.**
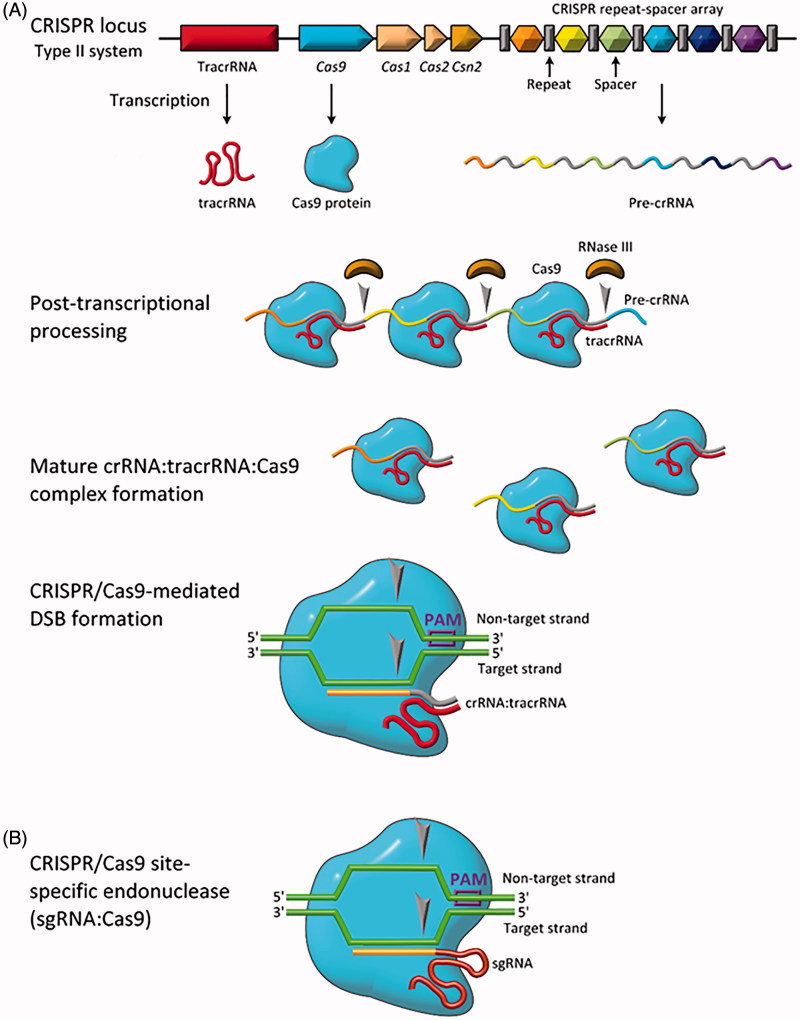
Biology of the type II CRISPR/Cas system. (A) Genomic representation of CRISPR/Cas9 along with relevant transcription/translation products. (B) Engineered CRISPR/Cas9 for site-specific gene editing (sgRNA:Cas9). Grey arrows indicate sites of single-stranded nucleotide breaks.

Type II CRISPR systems require only one protein, Cas9, to scan, bind and cleave the target DNA sequence (Makarova et al., 2011). Details of the Type II CRISPR/Cas9 system are shown in Figure 4(A). The genomic CRISPR locus is comprised of three components: the trans-activating CRISPR RNA (tracrRNA) gene, the Cas gene, and the CRISPR repeat and spacer sequences (Chylinski et al., 2014). These are transcribed into tracrRNA, Cas9 protein, and pre-crRNA. Following transcription, the tracrRNA and pre-crRNA are stabilized by Cas9 and base pair, and RNase III processes the pre-crRNA into crRNA by cleaving it at the repeat (Deltcheva et al., 2011). This dependence on RNase III likely explains why Type II systems are found in bacteria and not archaea, as RNase III is not found in archaea (Garrett et al., 2015). The crRNA:tracrRNA:Cas9 complex forms the active crRNA-guided endonuclease (Chylinski et al., 2013).

The Cas9:RNA complex randomly interrogates DNA in the cell, searching first for the appropriate protospacer adjacent motif (PAM), a short motif (5′-NGG-3′ for Cas9 from *Streptococcus pyogenes*) adjacent to the target sequence (Chylinski et al., 2013). Upon recognition of the PAM sequence, the Cas9:RNA complex unwinds the DNA from the first 10–12 nucleotides following the PAM sequence, termed the seed region (Szczelkun et al., 2014). If the interrogated DNA sequence matches the crRNA target sequence, the HNH nuclease domain of the Cas9 nuclease lobe cleaves the target strand while the RuvC-like nuclease domain of the Cas9 α-helical lobe cleaves the non-target strand (Anders et al., 2014; Jinek et al., 2014; Nishimasu et al., 2015). Single mismatches, and sometimes multiple mismatches, can be tolerated by the Cas9:RNA complex, with mismatches being more tolerated in regions downstream of the seed region (Cong et al., 2013; Sternberg et al., 2014).

## Type II CRISPR/Cas9 for gene editing

In view of the simplicity of the Type II CRISPR/Cas9 system, researchers first exploited use of CRISPR for gene editing using the Cas9 system from *S. pyogenes.* In 2012, Doudna and Charpentier showed the first use of CRISPR/Cas9 to introduce DSBs in target DNA (Jinek et al., 2012). Further, they showed that the duo-tracrRNA:crRNA units could be engineered into a single, truncated RNA chimera and still direct efficient DNA cleavage. As shown in Figure 4(B), this further simplified CRISPR/Cas9 into a two component system: a Cas9 protein and a single guide RNA (sgRNA). This simplicity makes the CRISPR/Cas9 system the most convenient, simple, and flexible tool for site-directed gene editing currently available.

In early 2013, only a few months after the publication of Jinek et al. (2012), three independent groups reported the use of CRISPR/Cas9 for gene editing. Cho et al. (2013) used the *S. pyogenes* Cas9 system (SpCas9) to edit human cells to incorporate GFP and RFP. Mali et al. (2013) engineered the SpCas9 system and cloned it into human cells, then performed multiplexed editing of target loci. Cong et al. (2013) engineered two different Type II CRISPR/Cas9 systems to introduce precise cleavage in human and mouse cell gene loci. Since these pioneering works, CRISPR/Cas9 has been implemented for gene editing in thousands of laboratories worldwide.

## CRISPR: beyond gene editing

In addition to site-specific gene editing, the DNA-binding properties of CRISPR/Cas9 may prove useful in other important applications. Qu et al. repurposed CRISPR into an RNA-guided platform for controlling gene expression by developing a catalytically-dead Cas9 enzyme (dCas9) that retained its capability to recognize and bind a target DNA sequence (Qi et al., 2013). Instead of cleaving the bound DNA, the dCas9 enzyme remained bound to the target DNA sequence, disrupting RNA polymerase or transcription factor binding. They showed that this system, termed CRISPR interference (CRISPRi), could repress expression of multiple genes simultaneously without altering the genome. They demonstrated this gene repression in both *Escherichia coli* and human cells. Shortly after reporting the gene interference CRISPR variant, this same group developed a gene expression/amplification CRISPR (CRISPR activation, CRISPRa) by creating a fusion protein of a dCas9 and a repeating peptide array transcription factor (Tanenbaum et al., 2014). Using these two tools together, genomic libraries of CRISPR inhibition and activation systems were created and used to screen sensitivity to a cholera-diphtheria toxin (Gilbert et al., 2014). Further, Mali et al. (2013) showed that adding functional RNA components to the sgRNA can also enhance transcriptional activity. Two copies of a MS2 RNA aptamer stem-loop sequence were added to the 3’ end of the sgRNA and used together with a Cas9–VP64 activation domain fusion, resulting in robust sequence-specific transcriptional activation. Finally, a system utilizing another catalytically dead Cas9 has been recently described by Gaudelli et al. (2017) for precise base editing. Using a seventh-generation evolved transfer RNA adenosine deaminase attached to dCas9, researchers demonstrated conversion of A*T base pairs to G*C base pairs in a targeted fashion at efficiencies of around 50% with few undesired mutations in human cells. As is the case with CRISPRi and CRISPRa, the Cas9 component is utilized for its precision targeting rather than for catalytic activity. Unlike with those activation and suppression systems, however, the result of this system is gene editing with no double-stranded breaks. As many diseases are caused by single point mutations, this application of CRISPR could prove to be one of the system’s most powerful gene editing tools.

The CRISPR system may also serve as a powerful tool for epigenetic studies, allowing for targeted manipulation of epigenetic markers to interrogate epigenetic and transcriptional control relationships. A fusion protein of dCas9 and acetyltransferase was developed by Hilton et al. (2015), catalyzing acetylation of histone H3 lysine 27 at target sites. They showed highly specific gene activation across the genome. Other epigenetic markers (e.g. methyl groups) may be modulated using this approach.

Inducible CRISPR systems were also developed. A photoactivated Cas9 was generated from a split of Cas9 fragments and photoinducible dimerization domains. In response to blue light, the CRISPR/Cas9 system performed gene sequence modification (Nihongaki et al., 2015). Editing activity was extinguished by removing the light source. A similar blue light activating system was developed for the epigenetic gene activator CRISPR system of Hilton et al. discussed above (Polstein & Gersbach, 2015). Chemically induced CRISPRs have also been created. Dow et al. developed a doxycycline-regulated Cas9 that allowed for inducible *in vivo* genome editing in adult mice (Dow et al., 2015).

The specific DNA binding function of CRISPR has also been repurposed to detect the location of genes within undisturbed nuclei of fixed cells (Deng et al., 2015) and living human cells (Ma et al., 2015). Termed CASFISH, dCas9 proteins were labeled with differently colored fluorophores and coordinated to a specific sgRNA. This allowed for multicolor detection of specific genomic loci with high spatial resolution and the assessment of DNA compaction. Unlike traditional fluorescent *in situ* hybridization, this technique avoided the need of heat treatment and chemicals that can distort the natural organization of the nucleus. It is important to note that fusing proteins to dCas9, however, does not always result in a functional fusion (Ledford, 2016).

Finally, though the CRISPR/Cas9 system has traditionally only been utilized to modify or otherwise interact with DNA substrates containing a PAM site, some recent cutting-edge work suggests that RNA with no PAM site can also be an active substrate for Cas9. Strutt and colleagues demonstrated that Cas9 subtypes II-A and II-C can recognize and cleave RNA in a directed manner utilizing RNA–RNA interactions independent of the presence of a PAM site in the target RNA molecule (Strutt et al., 2018). This cleavage protected *E. coli* cells from infection with bacteriophage MS2 particles, suggesting that Cas9 can provide cellular defense against both DNA and RNA viruses. This exciting work allows for the possibility of direct RNA targeting via CRISPR/Cas9, further expanding the scope of the system for practical applications.

## Factors affecting efficacy of the CRISPR/Cas9 system

While the CRISPR/Cas9 system has demonstrated great promise for site-specific gene editing and other applications, there are several factors that influence its efficacy which must be addressed, especially if it is to be used for *in vivo* human gene therapy. These factors include target DNA site selection, sgRNA design, off-target cutting, incidence/efficiency of HDR vs. NHEJ, Cas9 activity, and the method of delivery. As delivery remains the major obstacle for use of CRISPR for *in vivo* applications, efforts addressing other factors will be briefly summarized here. A comprehensive synopsis of existing delivery strategies and potential future delivery candidates will follow.

### Target DNA site selection and sgRNA design

A powerful advantage of the CRISPR/Cas9 system is the ability to target any ∼23-bp sequence that contains a PAM motif on either strand of DNA. This motif has been reported to occur every eight bps, on an average, for the SpCas9 PAM (Ramakrishna et al., 2014). Cas9 proteins from other species are being characterized and found to have differing PAM sequences. As an example, the PAM from *Neisseria meningitidis* Cas9 is reported to be 5′-NNNNGATT-3′ (Jiang et al., 2013; Ma et al., 2014). This provides even greater flexibility in target sequence selection, and this flexibility will increase as new Cas9 proteins with differing PAMs are identified. Additionally, directed evolution and structure-guided rational design has allowed for engineering of Cas9 variants with altered PAM sequence specificity (Kleinstiver et al., 2015; Anders et al., 2016; Hirano et al., 2016). As examples, the VGR, EQR, and VRER variants of SpCas9 have PAM sequences of 5′-NGAN-3′, 5′-NGAG-3′, and 5′-NGCG-3′, respectively, further reducing limits on genome target selection imposed by the PAM sequence.

Reports from several groups, however, have shown that target site selection and sgRNA design are not as simple as perhaps originally assumed. As mentioned previously, single- and multiple-base mismatches can be tolerated, with mismatches more tolerated at greater distances from the PAM (Fu et al., 2013; Hsu et al., 2013; Pattanayak et al., 2013; Wang et al., 2014; Doench et al., 2014; Moreno-Mateos et al., 2015; Xu et al., 2015). One report suggests that CRISPR/Cas9 may be less specific than ZFNs or TALENs due to the relatively shorter targeting sequence (Cradick et al., 2013). This contrasts with many reports that show no detectable off-target cleavage from CRISPR/Cas9 editing, with off-target effects being guide-RNA-specific (Cradick et al., 2013; Fu et al., 2013; Hsu et al., 2013; Pattanayak et al., 2013;Cho et al., 2014). Rational design of the sgRNA has therefore been the subject of a significant body of work resulting in many criteria and no simple rules. There are now many computational tools and software packages available that facilitate sgRNA design. However, caution is still needed, as shown in a recent study by Haeussler et al. (2016) that compared predictions from several sgRNA design tools with experimental results published in eight SpCas9 off-target studies. The authors showed evidence of algorithmic overfitting and the importance of using a model trained on data from the same guide RNA expression system.

### Off-target cutting

In addition to rational design of sgRNA, efforts to improve specificity and reduce off-target cutting have resulted in the design of mutant Cas9 systems. While Cas9 itself does not cause off-target effects – these exist solely due to the sgRNA – improvements to the Cas9 protein can limit these effects nonetheless. One mutant system disrupts the Cas9 protein so that it introduces only single-stranded DNA nicks. The nickase CRISPR/Cas9 is then used as a pair with one Cas9 binding to the forward DNA sequence and another Cas9 binding to the reverse DNA sequence flanking the target site. Only when binding in this configuration is a DSB formed through cooperative nicks. Off-target cutting results in only a single-stranded nick that is repaired with simple DNA ligases. The use of this system in mammalian cells reduced off-target cutting by three orders of magnitude with little to no reduction in on-target cutting efficacy (Mali et al., 2013; Ran et al., 2013; Cho et al., 2014).

ZFN and TALEN systems served as inspiration for another mutant Cas9 system, a fusion protein of inactive dCas9 and a *Fok*I nuclease dimer. Again, sgRNAs are designed to bind both the forward and reverse sequences flanking the target, and only when bound in this configuration will the *Fok*I nuclease dimers reconstitute into a functional *Fok*I and form a DSB (Guilinger et al., 2014; Tsai et al., 2014). While these approaches increase specificity and reduce off-target cutting, the number of potential target sites is lower due to PAM and other sgRNA design constraints. This system also significantly increased the size of the gene editing tool, providing greater constraints on *in vivo* delivery approaches. Another reported mutant Cas9 systems designed to reduce off-target effects include fusions of Cas9 with ZFPs or TALEs that can target nearly any genomic locus with improved precision (Bolukbasi et al., 2015). Cas9 mutants have also been designed to reduce non-specific DNA contacts by weakening binding of the target DNA strand (Kleinstiver et al., 2016) or the non-target DNA strand (Slaymaker et al., 2016) to Cas9 while maintaining robust on-target cleavage.

### Incidence/efficiency of HDR

The incidence of HDR-mediated DNA repair from DSBs is typically extremely low in mammalian cells. For example, Cas9-based gene editing in mice resulted in HDR repair efficiencies of 0.5–20%, while NHEJ-mediated repair occurred at 20–60% (Maruyama et al., 2015). Even in the presence of donor template DNA, NHEJ is the more frequent repair mechanism observed from CRISPR/Cas9 editing (Maruyama et al., 2015). Several approaches have emerged to increase HDR efficiency and suppress NHEJ, including use of small molecular inhibitors of NHEJ (Srivastava et al., 2012; Tomkinson et al., 2013; Robert et al., 2015; Vartak & Raghavan, 2015; Yu et al., 2015), gene silencing (Chu et al., 2015), cell cycle synchronization (Lin et al., 2014), and use of cell lines deficient in NHEJ components (Weinstock and Jasin, 2006). One of the most commonly used inhibitors, Scr7, targets the NHEJ component DNA ligase IV, and has been reported to increase efficiency of HDR from Cas9 editing by up to 19-fold (Srivastava et al., 2012; Chu et al., 2015; Vartak & Raghavan, 2015). While the use of Scr7 and other inhibitors have resulted in increased HDR-mediated gene editing efficiency, these inhibitors may have toxic effects on the host cells. Recent work to synchronize cells into late S and G_2_ phase, where HDR can occur, along with direct nucleofection of Cas9 ribonuclease complex, may prove a viable alternative to chemical suppression of NHEJ (Lin et al., 2014).

### Cas9 activity

Several Cas9 proteins from differing species have been identified and used for gene editing, including *Staphylococcus aureus* (SaCas9) (Ran et al., 2013), *Neisseria meningitidis* (NmCas9) (Hou et al., 2013), and *S. thermophiles* (St1Cas9) (Kleinstiver et al., 2015). Each has differing PAM sequences and variable activity. Thus, selection of a specific Cas9 ortholog may provide improved gene editing efficiency for a given target sequence and should be considered as part of gene editing system design.

In addition to the inherent activity of a given Cas9 protein, other factors have been shown to influence activity. For gene editing in eukaryotic cells, Cas9 must translocate into the nucleus. In these systems, the nuclear location signal (NLS) is connected to the Cas9 protein. Increasing access to the NLS by adding a 32 amino acid spacer between the NLS and Cas9 was shown to increase DNA cleavage activity (Shen et al., 2013). Increasing the relative concentration of sgRNA to Cas9 protein was also shown in increase on-target cutting activity, presumably by ensuring all Cas9 proteins formed the active ribonucleoprotein complex (Kim et al., 2014). However, excessive sgRNA was also shown to increase off-target effects (Fu et al., 2013).

Finally, in comparison with other enzymes, the activity of Cas9 is quite low, with a single turnover rate of ∼0.3–1.0 min^−1^(Jinek et al., 2012). And, once bound to the target DNA sequence, displacement of Cas9 from the DNA strand, even after DSB formation, is challenging – 1 nM Cas9 cleaved ∼2.5 nM plasmid DNA after 120 min (Jinek et al., 2012). Thus, Cas9 is less like a catalytic enzyme and more like a single-shot actuator. While this characteristic may be useful in some instances, such as gene activation/inhibition or short-lived activity for gene editing with lower off-target effects, it may be undesirable for other applications where catalytic activity is useful.

## CRISPR delivery systems

Here we will discuss the features of the most widely used systems for delivery of CRISPR/Cas9 components. Delivery can be broken into two major categories: cargo and delivery vehicle. Regarding CRISPR/Cas9 cargoes, there are three approaches that are commonly reported: (1) DNA plasmid encoding both the Cas9 protein and the guide RNA, (2) mRNA for Cas9 translation alongside a separate guide RNA, and (3) Cas9 protein with guide RNA (ribonucleoprotein complex). The delivery vehicle used will often dictate which of these three cargos can be packaged, and whether the system is usable *in vitro* and/or *in vivo*. As an example, Cas9 protein is positively-charged, but oligonucleotides and Cas9:sgRNA RNP are negatively charged (Sun et al., 2015). Additionally, considerations for how tightly controlled the overall concentration of Cas9 is must also be made; by introducing Cas9 DNA instead of protein, it becomes more difficult to ascertain precisely how many functional Cas9 units are present in the system at any given timepoint.

Vehicles used to deliver the gene editing system cargo (Table 1) can be classified into three general groups: physical delivery, viral vectors, and non-viral vectors. The most common physical delivery methods are microinjection and electroporation, while methods such as hydrodynamic delivery are currently under investigation. Viral delivery vectors include specifically engineered adeno-associated virus (AAV), and full-sized adenovirus and lentivirus vehicles. Especially for *in vivo* work, viral vectors have found favor and are the most common CRISPR/Cas9 delivery vectors. Non-viral vector delivery is not as prominent as viral-based delivery; however, non-viral vectors possess several advantages over viral vectors and are a bourgeoning area of research. Non-viral vector systems include systems such as lipid nanoparticles, cell-penetrating peptides (CPPs), DNA ‘nanoclews’, and gold nanoparticles. There are additionally many delivery technologies that have not been demonstrated in the literature as suitable to CRISPR/Cas9 delivery, though they appear to naturally lend themselves to the application. Four such technologies are streptolysin O, multifunctional envelope-type nanodevices (MENDs), lipid-coated mesoporous silica particles, and other inorganic nanoparticles.

**Table 1.** CRISPR delivery vehicles and their common features. Relatively difficulty is a subjective measure of how difficult the delivery vehicle is to utilize overall on a four-point scale, where one point is ‘few reagents, facile kit provided’ and four points is ‘requires expert in field with significant experimental experience’.

Delivery vehicle	Composition	Most common cargo	Capacity	Advantages	Limitations	Ease of use	Text refs
Microinjection	Needle	DNA plasmid; mRNA (Cas9 + sgRNA); Protein (RNP)	nM levels of Cas9 and sgRNA	Guaranteed delivery into cell of interest	Time-consuming; difficult; generally *in vitro* only	****	Yang et al. (2013), Horii et al. (2014), Chuang et al. (2017), Nakagawa et al. (2015), Crispo et al. (2015), Raveux et al. (2017), Sato et al. (2015), Ma et al. (2014), Niu et al. (2014), Wu et al. (2013), Long et al. (2014), Ross (1995)
Electroporation; nucleofection	Electric current	DNA plasmid; mRNA (Cas9 + sgRNA)	nM levels of Cas9 and sgRNA	Delivery to cell population; well-known technique	Generally *in vitro* only; some cells not amenable	*	Hashimoto & Takemoto (2015), Chen et al. (2016), Qin et al. (2015), Matano et al. (2015), Paquet et al. (2016), Ousterout et al. (2015), Schumann et al. (2015), Wu et al. (2015), Ye et al. (2014), Choi et al. (2014), Wang et al. (2014), Zuckermann et al. (2015), Kim et al. (2014)
Hydrodynamic delivery	High-pressure injection	DNA plasmid; Protein (RNP)	nM levels of Cas9 and sgRNA	Virus-free; low cost; ease	Non-specific; traumatic to tissues	**	Yin et al. (2014), Guan et al. (2016), Xue et al. (2014), Lin et al. (2014), Zhen et al. (2015), Dong et al. (2015)
Adeno-associated virus (AAV)	Non-enveloped, ssDNA	DNA plasmid	<5kb nucleic acid	Minimal immunogenicity	Low capacity	***	Yang et al. (2013), Long et al. (2016), Carroll et al. (2016), Platt et al. (2014), Hung et al. (2016), Swiech et al. (2015), Chew et al. (2016), Truong et al. (2015), Ran et al. (2015), Nelson et al. (2016), Tabebordbar et al. (2016), Esvelt et al. (2013)
Adenovirus	Non-enveloped, dsDNA	DNA plasmid	8kb nucleic acid	High efficiency delivery	Inflammatory response; difficult scaled production	***	Voets et al. (2017), Maddalo et al. (2014), Wang et al. (2015), Ding et al. (2014), Maggio et al. (2016), Li et al. (2015), Cheng et al. (2014)
Lentivirus	Enveloped, RNA	DNA plasmid	∼10kb, up to 18 kb nucleic acid	Persistent gene transfer	Prone to gene rearrangement; transgene silencing	***	Shalem et al. (2014), Wang et al. (2014), Naldini et al. (1996), Kabadi et al. (2014), Heckl et al. (2014); Roehm et al. (2016), Koike-Yusa et al. (2014), Ma et al. (2015), Zhang et al. (2016), Platt et al. (2014)
Lipid nanoparticles/ liposomes/lipoplexes	Natural or synthetic lipids or polymers	mRNA (Cas9 + sgRNA); Protein (RNP)	nM levels of Cas9 and sgRNA	Virus-free; simple manipulation; low cost	Endosomal degradation of cargo; specific cell tropism	**	Yin et al. (2016), Wang et al. (2016), Zuris et al. (2015), Horii et al. (2013), Sakuma et al. (2014), Schwank et al. (2013), Liu et al. (2014), Liang et al. (2015), Kennedy et al. (2014), Miller et al. (2017), Ebina et al. (2013)
Cell-penetrating peptides (CPPs)	Short amino acid sequences	Protein (RNP)	nM levels of Cas9 and sgRNA	Virus-free; can deliver intact RNP	Variable penetrating efficiency	**	Ramakrishna et al. (2014), Axford et al. (2017)
DNA nanoclew	DNA spheroid	Protein (RNP)	nM levels of Cas9 and sgRNA	Virus-free	Modifications for template DNA needed	****	Sun et al. (2015), Sun et al. (2014)
Gold nanoparticles (AuNPs)	Cationic arginine-coated AuNP	Protein (RNP)	nM levels of Cas9 and sgRNA	Inert; membrane-fusion-like delivery	Nonspecific inflammatory response	**	Mout et al. (2017), Lee et al. (2017)
iTOP	Hyperosmlality + transduction compound	Protein (RNP)	nM levels of Cas9 and sgRNA	Virus-free; high-efficiency	Non-specific; no *in vivo* use yet reported	***	D'Astolfo et al. (2015)
SLO	Bacterial pore-forming toxin	∼100kDa proteins and complexes	Unknown for CRISPR	Reversible pore formation; no impact on cell viability	Not yet proven with CRISPR	***	Sierig et al. (2003), Walev et al. (2001), Brito et al. (2008), Teng et al. (2017)
MENDs	Poly-lysine core, lipid coating, CPP decoration	Nucleic acids	Unknown for CRISPR	Customizable; readily modified for precise delivery	Not yet proven with CRISPR	****	Kogure et al. (2004), Nakamura et al. (2012)
Lipid-coated mesoporous silica NPs	Mesoporous Si coated with lipid	Small molecules and short RNA sequences	Unknown for CRISPR	Inert; easy modification with targeting moieties	Not yet proven with CRISPR	***	Liu et al. (2009), Du et al. (2014), Durfee et al. (2016), Gonzalez Porras et al. (2016), Mackowiak et al. (2013), Su et al. (2017), Wang et al. (2013)
Inorganic NPs	NPs of various compositions (carbon, silica)	Large proteins, nucleic acids	Unknown for CRISPR	Inert; used for similar applications	Not yet proven with CRISPR	**	Bates & Kostarelos (2013), Luo et al. (2014), Luo & Saltzman (2000)

## Physical delivery methods

### Microinjection

Microinjection is considered the ‘gold standard’ for introducing CRISPR components into cells, with efficiencies approaching 100% (Yang et al., 2013; Horii et al., 2014). In this method, either plasmid DNA encoding both the Cas9 protein and the sgRNA, mRNA encoding Cas9 and sgRNA, or Cas9 protein with sgRNA, can be directly injected into individual cells. Using a microscope and a 0.5–5.0 μm diameter needle, a cell membrane is pierced and cargoes are delivered directly to a target site within the cell. This process circumnavigates barriers associated with delivery through extracellular matrices, cell membranes, and cytoplasmic components. Further, microinjection is not limited by the molecular weight of the cargo, which is a significant limiting factor with viral vector delivery systems. This method also allows for the controlled delivery of known quantities of the cargo, improving control over off-target effects. Naturally, microinjection is best suited for *in vitro* and *ex vivo* work only, as the use of a microscope to target individual cells (and precisely inject cargoes to specific locations within them) precludes the use of microinjection in a true *in vivo* setting.

Nucleic acids are by far the most common cargo for microinjection delivery. There are three primary methods for injection of these components: (1) as DNA directly delivered to the cell nucleus, (2) as *in vitro*-transcribed mRNA molecules delivered to the nucleus, or (3) as *in vitro*-transcribed mRNA molecules delivered to the cytoplasm. These different methods have benefits and drawbacks. By placing the DNA encoding both Cas9 and the sgRNA into the nucleus, the cell is free to transcribe and translate the components. This method is preferred by some groups, such as Chuang et al. (2017) and Nakagawa et al. (2015), due to the ability to omit lengthy *in vitro* transcription reactions from the overall process. However, single-stranded DNA is prone to random integration into the host genome, which may disrupt genes, result in constitutive expression of Cas9, and lead to greater off-target effects. Even circularized plasmid DNA can undergo this phenomenon (Yang et al., 2013).

**Figure 5.**
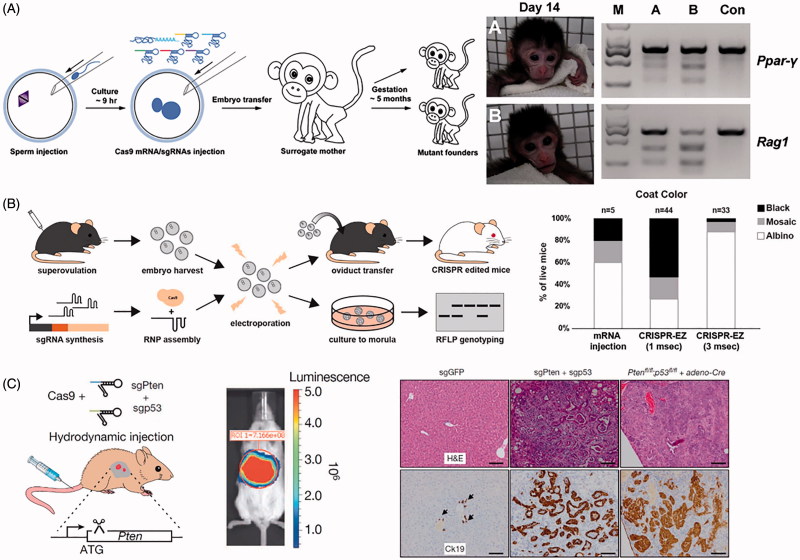
Physical methods for delivery of CRISPR. (A) Microinjection disrupting two genes (*Ppar-γ* and *Rag1*) in Cynomolgus monkeys from a single injection into one-cell-stage embryos. Photographs of Founder Monkeys A and B, PCR products of the targeted loci from genomic DNA of A and B, and a control wild-type Cynomolgus monkey (Con). Adapted with permission from Nui et al. (2014). Copyright 2014 Elsevier Inc. (B) Electroporation delivery of CRISPR RNP targeting genes impacting mice coat color (*Tyr*) followed by transfer to pseudopregnant mothers. Bar plot quantifies coat color phenotypes generated from microinjection and electroporation at 1 ms pulse length and 3 ms pulse length. Adapted with permission from Chen et al. (2016). Copyright 2016 American Society for Biochemistry and Molecular Biology. (C) Hydrodynamic injection of CRISPR into mice results in liver-specific targeting (see bioluminescence image of hydrodynamically injected luciferase plasmid), generating indel mutation of two tumor suppressor genes and oncogenes. The development of liver tumors can be seen in the hematoxylin and eosin (H&E) and cytokeratin 19 (Ck19)-stained micrographs. Adapted with permission from Xue et al. (2014). Copyright 2014 Macmillan Publishers Ltd: Nature.

When delivering mRNA, the ideal case is to deliver the sgRNA directly to the nucleus and the Cas9-encoding mRNA to the cytoplasm, facilitating translation and shuttling of Cas9 to the nucleus. Unfortunately, microinjection is a technically challenging and laborious process, making two different microinjections into a single cell impractical. Further, two microinjections, even when separated by several hours, typically results in non-viable cells (Yang et al., 2013). Therefore, microinjections of CRISPR mRNA components often occurs directly into the cytoplasm of the cell; for some examples see Crispo et al. (2015), Raveux et al. (2017), and Sato et al. (2015). This method has the advantage of putting the Cas9 mRNA directly into the cytoplasm, where it can be translated by the cell. sgRNA in the cytoplasm is then bound by Cas9 while being shuttled into the nucleus, allowing for modification of the host DNA. The vast majority of studies using microinjection to deliver CRISPR use this approach, including simultaneous knock-out of four genes from a single injection into rat zygotes (Ma et al., 2014), disruption of two genes in cynomolgus monkeys from a single injection into one-cell-stage embryos (see Figure 5(A)) (Niu et al., 2014), correction of a cataract-causing mutation in mice (Wu et al., 2013), and correction of a Duchenne muscular dystrophy (DMD)-causing mutation in mice (Long et al., 2014). With some exceptions, microinjection of CRISPR/Cas9 RNA components into cells results in a finite duration of action of the system, owing to the natural decay of mRNA within eukaryotic cells (Ross, 1995). This is often desirable as it reduces off-target effects.

Microinjection is also the most commonly used method for generating animal models. Injection of the gene editing cargo into zygotes allows for efficient germline modification. In addition, there is evidence that injection of Cas9 mRNA and sgRNA into the zygote cytoplasm is the most efficient method for yielding normal embryos and full-term mouse pups harboring the desired modification (Horii et al., 2014). Microinjection can also be useful for CRISPRa and CRISPRi to provide transient up- or down-regulation of a specific gene within the genome of a mature cell. Microinjection is a well-established technology and its use is widespread, as evidenced by the ability to custom-order microinjected mouse zygotes from facilities such as the Genome Modification Facility at Harvard University (https://gmf.fas.harvard.edu/talen-or-crispr-microinjection).

### Electroporation

One of the long-standing physical methods for delivery of gene editing tools into a population of cells is electroporation. This technique utilizes pulsed high-voltage electrical currents to transiently open nanometer-sized pores within the cellular membrane of cells suspended in buffer, allowing for components with hydrodynamic diameters of tens of nanometers to flow into the cell. Electroporation is less dependent on cell type than other delivery techniques and can efficiently transfer cargo into cells that are traditionally difficult to manipulate. Electroporation is most commonly used in an *in vitro* setting, though as with microinjection, *ex vivo* applications are also valid. Owing to the oftentimes-large amounts of voltage needed to be applied across cell membranes, however, electroporation is typically not suitable for *in vivo* applications.

There are many published methods for electroporation of mammalian cells. While these protocols can provide a starting point, mammalian cells are often quite sensitive to precise voltages and current application times. This contrasts with bacterial cells, which are often more tolerant of electroporation. This problem becomes even more prominent when studying zygotes rather than immortalized cell lines.

Several groups have developed technological solutions to increase the prominence of electroporation within the CRISPR/Cas9 community. For example, Hashimoto and Takemoto (2015) built a custom electroporation chamber for 40–50 zygotes which allowed them to achieve both very high levels of CRISPR/Cas9 entry into cells and viable embryos. Other groups have used more standard electroporation cuvettes and methods to deliver CRISPR/Cas9 components with high efficiency to zygotes (Figure 5(B)) (Qin et al., 2015; Chen et al., 2016). In other examples, electroporation was used to deliver plasmid DNA encoding both Cas9 and mRNA to generate colorectal cancer models from Cas9-engineered human intestinal organoids (Matano et al., 2015), generate an early-onset Alzheimer’s disease model in human cells (Paquet et al., 2016), and correct mutations that cause DMD (Ousterout et al., 2015). Electroporation for delivery of Cas9:sgRNA ribonucleoprotein (RNP) to primary human cells has also been reported (Kim et al., 2014; Schumann et al., 2015). The use of RNP was shown to reduce off-target effects versus plasmid transfection and be less stressful on the cells, producing two-fold more embryonic stem cell colonies than with plasmid transfection (Kim et al., 2014).

A specialized electroporation method designed to deliver cargoes directly into the nuclei of mammalian cells has also been used to deliver CRISPR/Cas9. Termed nucleofection, this technique does not require breaking down the nuclear envelope, or cells in a state of division, for cargo to enter the nucleus. Plasmid DNA encoding Cas9 and sgRNA has been delivered via nucleofection to correct a cataract-causing mutation in mouse spermatogonial stem cells (Wu et al., 2015), confer resistance to HIV infection by adding the natural CCR5Δ32 mutation to human cells (Ye et al., 2014), generate lung cancer cell models in human lung epithelial cells (Choi & Meyerson, 2014), and cure latent herpesvirus infection by Cas9-based cleavage and destruction of latent viral genomes (Wang & Quake, 2014).

The use of electroporation and CRISPR/Cas9 to edit genes *in vivo* was also recently reported. Zuckermann et al. (2015) developed a model of a childhood malignant brain cancer, Sonic hedgehog medulloblastoma, via *in utero* electroporation of a developing mouse. Plasmids encoding both Cas9 and sgRNA were injected into the embryo cerebral ventricular zone, followed by electroporation using forceps-like electrodes.

Because of the attractiveness of high-throughput and high-efficiency CRISPR/Cas9 transformation utilizing commonly available laboratory resources, electroporation/nucleofection will likely continue to be used and refined as a major technique to efficiently deliver CRISPR/Cas9.

### Hydrodynamic delivery

Hydrodynamic delivery is an i*n vivo* delivery technique that involves rapidly pushing a large volume (8–10% body weight) solution containing the gene editing cargo into the bloodstream of an animal, typically using the tail vein in mice. As blood is incompressible, the large bolus of liquid results in an increase in hydrodynamic pressure that temporarily enhances permeability into endothelial and parenchymal cells, allowing for cargo not normally capable of crossing a cellular membrane to pass into cells. This includes naked DNA plasmids and proteins. Delivery of cargo using this method is significantly enriched in the liver, but also includes cells of the kidneys, lung, muscles, and heart. Hydrodynamic delivery is attractive because it is technically simple and does not require any exogenous delivery components to successfully introduce gene editing components into cells. Hydrodynamic delivery is typically used for *in vivo* applications only, as the premise of delivery relies on transiently increasing the pressure in a closed system and forcing cargo through otherwise-impermeable barriers.

Yin et al. (2014) demonstrated successful delivery of DNA plasmid encoding Cas9 and sgRNA to liver cells using hydrodynamic delivery, resulting in *in vivo* correction of the *Fah* mutation in mouse hepatocytes modeling hereditary tyrosinemia. Although initial delivery efficiency was only one in 250 liver cells, the liver’s regenerative capacity allowed for the expansion of the modified cells and phenotype rescue. Soon after, Guan et al. also used hydrodynamic delivery of naked plasmid DNA encoding CRISPR/Cas9 components to a mouse model of hemophilia B. Again, targeting the liver, they showed restored hemostasis in treated mice (Guan et al., 2016). They also showed that an adenovirus (AdV) delivery system resulted in higher corrective efficiency, but no therapeutic effects due to severe hepatic toxicity, presumably a result of the high immunogenicity of the viral vector. Other examples of hydrodynamic injection of plasmid DNA encoding Cas9 and sgRNA include indel mutation of two tumor suppressor genes and oncogenes resulting in generation of liver tumors in mice (Figure 5(C)) (Xue et al., 2014), inhibiting hepatitis B virus (HBV) replication and gene expression in HBV-infected mice (Lin et al., 2014; Zhen et al., 2015), and specific targeting of the HBV genome in the nucleus of HBV-infected mice, showing the potential of CRISPR/Cas9 as a therapeutic against chronic HBV infection (Dong et al., 2015).

Despite these successes, hydrodynamic delivery is not currently being considered for clinical applications. The process of hydrodynamic delivery can be quite traumatic, resulting in potential physiological complications, including cardiac dysfunction, elevated blood pressure, and liver expansion (Suda et al., 2007; Bonamassa et al., 2011). It is relatively easy to cause accidental mortality with this method. Also, as discussed previously, transfection rates are very low, and only certain cell types are amenable to successful delivery.

## Viral vector delivery methods

### Adeno-associated virus (AAV)

AAV, of the *Dependovirus* genus and *Parvoviridae* family, is a single stranded DNA virus that has been extensively utilized for gene therapy (Daya and Berns, 2008; Samulski and Muzyczka, 2014). AAV is an excellent vehicle for gene therapy for many reasons. AAV is not known to cause or relate with any diseases in humans. There is also a wide range of known serotypes which allow for infection of a multitude of cells with different specificities. The virus itself is able to efficiently infect cells while provoking little to no innate or adaptive immune response or associated toxicity, at least upon first treatment with a serotype (Daya and Berns, 2008). Immune responses are eventually seen to the capsid, sometimes even causing CD-8 T-cell toxicity (Samulski and Muzyczka, 2014). However, owing to the many serotypes of AAV with broad tropism, it is often possible to evade the problem of immune response to AAV should it arise. Finally, unlike some other methods, the use of AAV for gene therapy provides a persistent source of the provided DNA, as AAV-delivered genomic material can exist indefinitely in cells either as exogenous DNA or, with some modification, be directly integrated into the host DNA (Deyle and Russell, 2009). This can, of course, be either advantageous or disadvantageous depending on the desired goals of a specified modification. AAV particles can see application in *in vitro*, *ex vivo*, and *in vivo* work, making them highly versatile delivery vehicles.

CRISPR/Cas9 AAV particles are typically created in HEK 293 T cells. Once particles with specific tropism have been created, they are used to infect the target cell line much in the same way that native viral particles do. This is what ultimately allows for persistent presence of CRISPR/Cas9 components in the infected cell type, and what makes this version of delivery particularly suited to cases where long-term expression is desirable.

**Figure 6.**
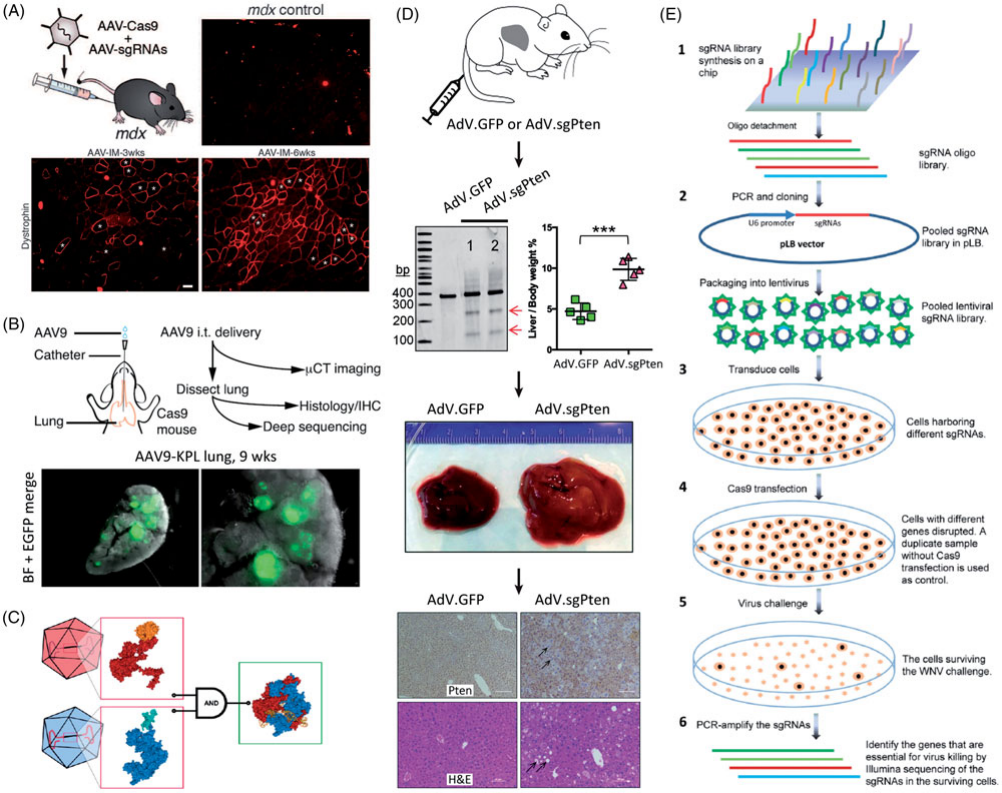
Viral vector methods for delivery of CRISPR. (A) AAV delivery of Cas9 and sgRNAs disrupting mutations in the Dmd gene in adult *mdx* mice, resulting in improvement of muscle biochemistry and function. Adapted with permission from Long et al. (2016). Copyright 2016 American Association for the Advancement of Science. (B) AAV intratracheal instillation delivery of sgRNAs in Cre-dependent Cas9 knock-in mice, resulting in lung adenocarcinoma (EGFP-positive tumors). Adapted with permission from Platt et al. (2016). Copyright 2014 Elsevier Inc. (C) A split Cas9 system in which the Cas9 C-terminal is packaged into one AAV vector and the Cas9 N-terminal is packaged into a second AAV vector. Reconstitution results in a fully functioning Cas9. Reprinted from Truong et al. (2015). Copyright 2014 The Authors (CC BY license). (D) AdV delivery of Cas9 and sgRNA targeting the *Pten* gene in mouse liver resulting in *Pten* mutation (see arrows by gel), and massive hepatomegaly and features of NASH in infected livers. Immunohistochemistry shows loss of Pten staining (arrows) one month after AdV infection; H&E stained micrographs show sections of steatosis (lipid accumulation, arrows) four months post infection. Adapted with permission from Wang et al., 2015. Copyright 2015 Mary Ann Liebert, Inc. Publishers. (E) Schematic of a lentivirus and CRISPR-based gene library functional screen used to identify the genes essential for West-Nile-virus-induced cell death. Reprinted from Ma et al. (2015). Copyright 2015 The Authors (CC BY license).

With specific regard to CRISPR/Cas9, AAVs are typically utilized as a delivery system in one of the four ways. In the first, SpCas9 and sgRNA are packaged directly onto one DNA plasmid vector and delivered via one AAV particle. While this is within the realm of technical possibility, the SpCas9 and sgRNA are roughly 4.2kB in size, and the overall size of AAV (∼20 nm) only allows for ∼4.5–5 kb of genomic material to be packaged within it (Wu et al., 2010). This makes consistent packaging of this construct challenging, and it is also extremely difficult to include other elements (such as reporters, fluorescent tags, multiple sgRNAs, or DNA templates for HDR) to help ensure successful delivery of CRISPR/Cas9 components to cells and meet desired gene editing objectives. This has been accomplished before by Long et al. (2016) who utilized a mini-cytomeglovirus promoter/enhancer with SpCas9 to correct DMD-causing mutations in mice (Figure 6(A)). AAVs were delivered by intraperitoneal, intramuscular, or retro-orbital injection and resulted in muscle function enhancement to varying degrees.

In another approach, AAVs were used to deliver sgRNAs into cells that were previously engineered to express Cas9. Carroll et al. (2016) used microinjection to transfect mouse embryos to express Cas9 in cardiomyocytes and then used AAVs to deliver sgRNAs, resulting in a cardiovascular research model that allows for rapid introduction of indels in heart tissue. Platt et al. developed Cre-dependent Cas9 knock-in mice and used AAVs to deliver sgRNAs, inducing loss-of-function mutations in tumor suppressing genes and gain-of-function mutations in proto-oncogens (see Figure 6(B)). This resulted in the generation of lung adenocarcinoma (Platt et al., 2014).

Many groups have reported success packaging the SpCas9 and sgRNA into two separate AAV particles and using them for co-infection (Swiech et al., 2015; Hung et al., 2016). This has the added benefit of increasing the overall size of the constructs that can be used. However, this naturally adds more complexity than exists with a single vector. Multiple tags (one for each particle) can be employed to preemptively screen cells for co-infection. In a similar approach, a split Cas9 system has been developed in which the Cas9 C-terminal is packaged into one AAV vector and the Cas9 N-terminal is packaged into a second AAV vector (Figure 6(c)) (Truong et al., 2015; Chew et al., 2016). Reconstitution of the two Cas9 halves results in a functional Cas9 with editing efficiency comparable to the native Cas9, allowing for the use of larger overall Cas9 variants with AAV particles.

The most recently developed AAV CRISPR/Cas9 delivery method was reported by Ran et al. (2015), and it uses a version of Cas9 from *S. aureus* rather than *Streptococcus pyrogenes* (designated SaCas9). This version of Cas9 is roughly 70% the size of SpCas9 while retaining the same potent cutting ability. This results in the ability to use a single vector, but the decrease in size leaves ∼1 kB of ‘free space’ within the AAV particle. This is often enough to include multiple different tags and markers in one particle. Groups have used of SaCas9 in AAV vectors to target the cholesterol regulatory gene Pcsk9 (Ran et al., 2015) and disrupt mutations in the DMD gene in adult mice (Nelson et al., 2016; Tabebordbar et al., 2016). Shorter Cas9 variants from *Streptococcus thermophilus* (Cong et al., 2013) and *Neisseria meningitidis* (Esvelt et al., 2013) have also been used for gene editing and may be good candidates for AAV delivery of CRISPR/Cas9. It should be noted, however, that shorter Cas9 variants identified to date have longer PAM sequences and thus greater limitations on sequences available for targeting.

### Lentivirus (LV) and adenovirus (AdV)

While LVs and AdVs are clearly distinct, the way they are utilized for delivery of CRISPR/Cas9 components is quite similar. In the case of LV delivery, the backbone virus is a provirus of HIV (Naldini et al., 1996); for AdV delivery, the backbone virus is one of the many different serotypes of known AdVs. As in the case of AAV, these are plentiful, and finding a useful AdV to a desired target is relatively facile. The serotype most commonly used is AdV type 5. LV is particularly useful because it can be pseudotyped with other viral proteins, such as the G-protein of vesicular stomatitis virus. In doing so, the cellular tropism of the LV can be altered to be as broad or narrow as desired. And, to improve safety, second- and third-generation LV systems split essential genes across three plasmids, significantly reducing the likelihood of accidental reconstitution of viable viral particles within cells. Both LV and AdV can infect dividing and non-dividing cells; however, unlike LV, AdV does not integrate into the genome. This is advantageous in the case of CRISPR/Cas9-based editing for limiting off-target effects. As is the case with AAV particles, both LV and AdV can be used in *in vitro*, *ex vivo*, and *in vivo* applications, which eases both efficacy and safety testing.

In terms of mechanism, this class of CRISPR/Cas9 delivery is like AAV delivery described above. Full viral particles containing the desired Cas9 and sgRNA are created via transformation of HEK 293 T cells. These viral particles are then used to infect the target cell type. The biggest difference between LV/AdV delivery and AAV delivery is the size of the particle; both LVs and AdVs are roughly 80–100 nm in diameter. Compared with the ∼20 nm diameter of AAV, larger insertions are better tolerated in these systems. When considering CRISPR/Cas9, additional packaging space for differently-sized Cas9 constructs or several sgRNAs for multiplex genome editing is a significant advantage over the AAV delivery system.

Many groups are currently using AdV or LV vectors for delivery of CRISPR/Cas9 components. Voets et al. (2017) recently used an AdV vector to inactivate genes in normal human lung fibroblasts and bronchial epithelial cells, wherein they reported efficient silencing of genes at MOIs of AdV as low as 20. Additionally, Kabadi et al. (2014) created a unique lentiviral CRISPR/Cas9 system via Golden Gate synthesis. Their construct allowed for the expression of one Cas9 and four different sgRNAs, each under the control of a different promoter, to allow for the editing of several different types of human cells. The work done by these two groups, especially on primary human cells, allows for exciting possibilities for the use of these delivery systems *in vivo*.

Maddalo et al. (2014) reported the generation of a model of *Eml4-Alk* oncogene-driven lung cancer in adult mice by intratracheal instillation of AdV-delivered CRISPR/Cas9. Wang et al. (2015) used AdV delivery of Cas9 and sgRNA to target *Pten*, a gene involved in the liver disease non-alcoholic steatohepatitis (NASH) as shown in Figure 6(D). Four months post treatment, *Pten* gene-edited mice showed massive hepatomegaly and features of NASH. Importantly, in addition to displaying AdV vector-associated immunotoxicity in the liver, humoral immunity against SpCas9 was detected, as was a potential SpCas9-specific cellular immune response. This indicates the importance of also studying the immunogenicity of specific Cas9s for *in vivo* delivery of CRISPR.

The use of AdV for CRISPR/Cas9 delivery has been reported targeting loss-of-function *PCSK9* mutation in mouse liver (Ding et al., 2014), *in vitro* partial restoration of muscle function in a DMD model mice (Maggio et al., 2016), resistance to HIV-1 infection of primary CD4 + T-cells by adding the cell membrane receptor *CCR5*Δ32 variant (Li et al., 2015), and tissue-specific gene knockout in mouse liver (Cheng et al., 2014).

Examples of LV delivery of CRISPR/Cas9 include modification of up to five genes using a single LV to deliver plasmid DNA encoding Cas9, sgRNA and a fluorescent marker to develop a mouse model of acute myeloid leukemia (Heckl et al., 2014), target of herpes simplex virus-1 genome regions essential to viral protein expression during early and late phases of viral infection/reinfection to suppress infection and prevent new infection (Roehm et al., 2016), and the use of LV to mutate genes in mouse primary immune cells (Platt et al., 2014).

Leveraging the integration ability of LV, this system has also been used to create gene libraries for studying mechanisms of disease. Wang et al. (2014) used LV to deliver a pool of 73,000 sgRNAs to two human cells lines that had been previously engineered to express Cas9. Shalem et al. (2014) delivered 64,751 unique sgRNA sequences by LV to human cancer cells, and Koike-Yusa et al. (2014) delivered 87,897 sgRNAs targeting 19,150 protein coding genes in mouse embryonic stem cells, both again constitutively expressing Cas9. These, and other similar studies, will enable identification of new therapeutic targets and the design of next-generation drugs. For example, genomics screening using CRISPR/Cas9 and LV was used to identify the genes essential for West-Nile-virus-induced cell death (Figure 6(e)) (Ma et al., 2015) and define a signal peptide pathway required by flaviviruses (Zhang et al., 2016).

However, there are drawbacks to LV or AdV delivery systems, as typical AdVs and LVs elicit strong immune responses (Follenzi et al., 2007; Ahi et al., 2011). In addition, although care is taken to make the HIV provirus as integration-deficient as possible (Chen and Goncalves, 2016) and AdVs are naturally very low integrators into the cell genome, it is not currently possible to completely eliminate the chances of integration into the host. Additionally, although steps can be taken to make this integration targeted, one cannot guarantee that the viral payload goes to the same precise location every time. This can result in an increase of expression and off-target effects, or even potential damage to the cell if the insertion randomly occurs within an important cellular protein (Bestor, 2000; Papapetrou and Schambach, 2016). Care must always be taken with LVs and AdVs when utilizing them for genome editing.

## Non-viral vector delivery vehicles

### Lipid nanoparticles/liposomes

Lipid nanoparticles have long been used as delivery vehicles for a wide range of different molecules to cells and have demonstrated popularity for nucleic acid delivery. Nucleic acids are typically unstable outside of cells, and owing to their highly anionic nature, they do not easily pass through the cell membrane. However, by encapsulating nucleic acids within typically very cationic liposomes, they can be delivered to cells with relative ease. Lipid nanoparticles do not contain any viral components, which helps minimize safety and immunogenicity concerns. They can also, like viral particles, be utilized *in vitro*, *ex vivo*, and *in vivo*, allowing for extensive testing on a variety of scales of cell populations.

When used to deliver CRISPR/Cas9 components, there are two main approaches to the use of lipid nanoparticles: delivering Cas9 and sgRNA genetic material (either plasmid DNA or mRNA) or delivering Cas9:sgRNA RNP complexes. If delivering Cas9 mRNA and sgRNA, this method is functionally like microinjection in result (Yin et al., 2016). However, several groups have shown good success with the use of Cas9:sgRNA RNP complexes (Zuris et al., 2015; Wang et al., 2016). CRISPR/Cas9 seems to be particularly well-suited to this type of delivery because Cas9 and the sgRNA as a ribonucleoprotein complex are highly anionic. This allows them to be packaged utilizing approaches typically employed for delivering nucleic acids.

**Figure 7.**
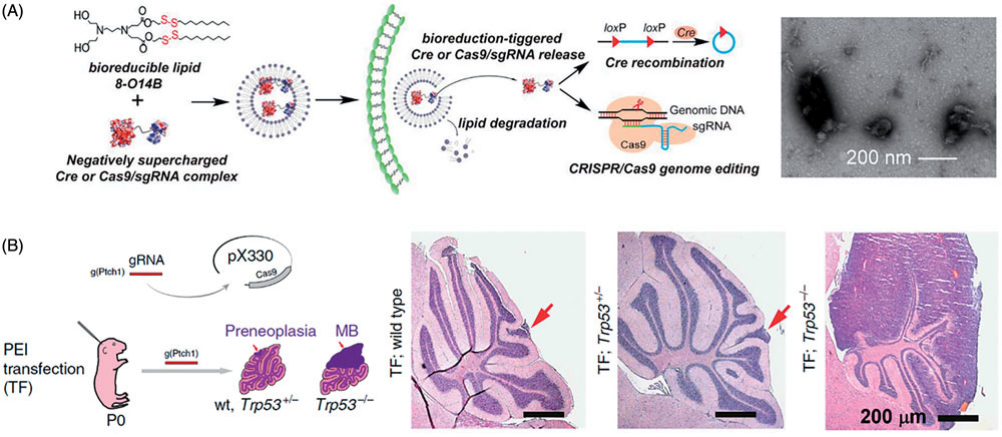
Lipid nanoparticle and polyplex delivery of CRISPR. (A) Combining bioreducible lipid nanoparticles and anionic Cas9:sgRNA complexes drives the electrostatic self-assembly of nanoparticles (see TEM micrograph of 3-O14B/Cas9:sgRNA nanoparticles) for potent protein delivery and genome editing. Adapted with permission from Wang et al. (2016). Copyright 2017 National Academy of Sciences. (B) Microinjection of PEI with Cas9- and sgRNA-encoding plasmid DNA into mouse brain directed against the *Ptch1* locus to generate a malignant brain tumor model. Compare the wild type and *Trp53*± H&E stained micrographs (arrows indicate small lesions encompassing only one cerebellar folium) with the tumor from the *Trp53*^−/−^ condition (MB = medulloblastoma). Adapted from Zuckermann et al. (2015). Copyright 2015 The Authors (CC BY license).

There are substantial drawbacks for delivery of CRISPR/Cas9 components via lipid nanoparticle. First, there are both external and internal barriers that must be considered. Once the nanoparticle has passed through the surface of the cell, it is typically encased within an endosome. Encased contents can very rapidly be directed by the cell into the lysosomal pathway, causing the degradation of all lysosome contents. Therefore, the cargo must escape the endosome. Also, if the Cas9:sgRNA complex can escape the endosome, it must also translocate to the nucleus, which can also be a potential point of failure. Because of this, it is rare to see particularly high efficacies when delivery CRISPR/Cas9 components via lipid nanoparticles. While Wang et al. (2016) could achieve ∼70% *in vitro* modification efficiency in cells (see Figure 7(A)), that only came after an intense screen to determine the most optimal lipids with which to construct their liposomes for their system. Finally, lipid nanoparticles are like virus particles in that the nature and size of the cargo, along with the target cell type, highly affect transfection efficiency and the types of lipids that are appropriate or useful in the system.

Perhaps the most commonly used lipid nanoparticle system is the commercially available Lipofectamine. Lipofectamine is a cationic liposome formulation that complexes to negatively charged nucleic acids, allowing fusion of the complex with negatively-charged cell membranes and endocytosis. Lipofectamine has been used to deliver Cas9- and sgRNA-encoding plasmid DNA to human pluripotent stem cells to generate a model for Immunodeficiency, Centromeric region instability, Facial anomalies syndrome (ICF) syndrome with 63% transfection efficiency (Horii et al., 2013), transfect human cells with an all-in-one expression cassette with up to seven sgRNAs and a Cas9 nuclease/nickase (Sakuma et al., 2014), correct the cystic fibrosis transmembrane conductor receptor locus in cultured intestinal stem cells of cystic fibrosis patients (Schwank et al., 2013), introduce modular ‘AND’ gate circuits based on CRISPR/Cas9 that detects bladder cancer cells, inhibits bladder cancer cell growth, induces apoptosis, and decreases cell motility (Liu et al., 2014), and deliver Cas9:sgRNA RNP *in vivo* to modify the hair cells within mouse inner ear (Zuris et al., 2015).

In an intriguing study, Liang et al. (2015) compared the transfection efficacy of three lipofectamine formulations and electroporation against eleven cell lines. They also compared different gene editing cargoes: plasmid DNA, Cas9 mRNA with sgRNA, and Cas9:sgRNA RNP. They showed greater efficiencies for electroporation transfection versus lipofectamine, and lower off-target effects using RNP over plasmid DNA or mRNA cargo.

Because of their lack of viral components, there will always be interest in improving lipid nanoparticles to deliver CRISPR/Cas9 components. This improvement process can come through the screening of better lipid carriers, as above; better decorations on the liposome surface to help target particles to specific cells or tissues, avoid immune system detection, and facilitate endosomal escape; and improved packaging of CRISPR/Cas9 components, increasing the odds of some subset of packaged molecules to be appropriately delivered.

### Lipoplexes/polyplexes

Delivery of CRISPR/Cas9 gene editing components has been reported using other nanocomplexes that generally rely on electrostatic interactions. A common approach is the use of the commercially available FuGENE-6 reagent, a non-liposomal solution containing lipids and other proprietary components. Kennedy et al. used FuGENE-6 to deliver Cas9 and sgRNA encoding plasmid DNA, inactivating human papillomavirus E6 or E7 gene in cervical carcinoma cells, resulting in cell-cycle arrest and eventual cell death (Kennedy et al., 2014). The synthesis and development of zwitterionic amino lipids (ZALs) was reported by Miller et al. (2017). ZALs were complexed with Cas9 mRNA and sgRNA, forming nanoparticles with ∼15 nm diameter which showed effective transfection in mice, accumulating primarily in the liver, kidney, and lungs. Another common and commercially available technique, calcium phosphate transfection, utilizes Ca^2+^ molecules to induce precipitation of DNA/Ca^2+^ microcomplexes. These complexes strongly bind to the negatively charged cell membrane and induce endocytosis into the cell. Ebina et al. (2013) used calcium phosphate transfection to deliver plasmid DNA encoding Cas9 and sgRNA into latent HIV-1-infected human 293 T cells. The CRISPR construct targeted the provirus genome, blocking expression of viral components and removing internal viral genes from the host cell chromosome.

Other common polymeric vectors for DNA delivery are polyethenimine (PEI) and poly(l-lysine) (PLL). Branched PEI have high charge density, facilitating efficient plasmid DNA packing, and pH-buffering ability which enables escape from endosomes. However, branched PEI is cytotoxic. Therefore, a balance between the desirable properties of branched PEI and the less toxic linear PEI must be struck for effective transfection. Zuckermann et al. (2015) reported the use of PEI to deliver Cas9- and sgRNA-encoding plasmid DNA into mouse brains to generate a malignant brain tumor model (Figure 7(B)). PEI was also used to deliver CRISPR plasmid DNA to inhibit HBV replication and gene expression in HBV-infected mice (Zhen et al., 2015). PLL has been used to complex with Cas9 plasmid DNA, forming a multifunctional envelope-type nanodevice (MEND), described in the Emerging Delivery Technologies section of this review.

### Cell-penetrating peptides (CPPs)

CPPs are generally short stretches of amino acids that are polycationic, amphipathic, or non-polar in nature. Each class of CPPs can facilitate uptake of different types of proteins into different cell types, and often different combinations of CPPs and the desired molecule for uptake will result in different uptake levels. CPPs can be used for *in vitro* and *ex vivo* work quite readily, and extensive optimization for each cargo and cell type is usually required. Because of the level of detail required for this optimization, CPPs are not generally currently utilized to deliver components *in vivo*. In the specific case of CRISPR/Cas9, the CPPs are usually covalently attached to the Cas9 protein directly, which is then complexed with the sgRNA and delivered to cells. Some work with CPPs and CRISPR/Cas9 was accomplished as early as 2014 by Ramakrishna et al. (2014), who showed separate delivery of CPP–Cas9 and CPP–sgRNA to multiple human cell lines. However, most reports are quite recent, such as the work done by Axford et al. (2017) in which the authors demonstrated cellular and sub-cellular localization of CPP-delivered CRISPR/Cas9 RNP using confocal microscopy.

Typically, CPPs show low efficiency of the desired targeted mutation in cells – usually around 10–20%. As shown above, however, naked plasmids can achieve tangible long-term effects with an efficiency rate of just 0.4%. As CPPs are roughly 40-fold more efficient than transfection from bare plasmids, CPPs are a serviceable method for delivery of CRISPR/Cas9 components to cells. This method requires a fair amount of investment, however, as efficiencies of the CPP themselves to penetrate cellular membranes vary with both attached cargo and cell type. The same challenges of translocating the Cas9:sgRNA complex into the nucleus once it is within the cell must also be overcome.

### DNA nanoclew

**Figure 8.**
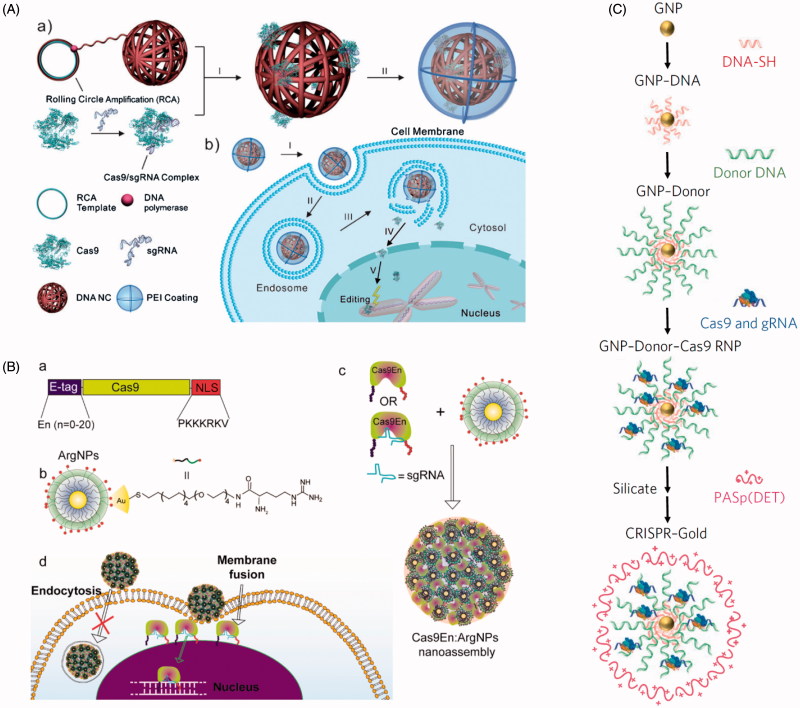
Nanomaterial delivery vehicles for CRISPR delivery. (A) DNA ‘nanoclew’ loaded with Cas9:sgRNA RNP via Watson-Crick base pairing, followed by coating with PEI to improve endosomal escape. Reprinted with permission from Sun et al. (2015). Copyright 2015 John Wiley & Sons. (B) Arginine-modified gold nanoparticles (ArgNPs, positively charged) interact with multiple Cas9:sgRNA RNPs engineered with an E-tag to form a local negatively charged region, forming a nanoassembly that delivers Cas9 via membrane fusion. Reprinted with permission from Mout et al. (2017). Copyright 2017 American Chemical Society. (C) Synthesis of AuNPs engineered to complex with multiple Cas9:sgRNA RNPs, followed by coating in silica and the endosomal disruptive polymer PASp(DET). Adapted with permission from Lee et al. (2017). Copyright 2017 Macmillan Publishers Ltd: Nature Biomedical Engineering.

A DNA ‘nanoclew’ is a unique technology for CRISPR/Cas9 component delivery. Developed by Sun et al. (2014), a DNA nanoclew is a sphere-like structure of DNA that has been compared with a ball of yarn. The nanoclew is synthesized by rolling circle amplification with palindromic sequences that aide in the self-assembly of the structure. The sphere can then be loaded with a payload – Sun et al. originally used doxorubicin – and the payload can be specifically triggered for release by certain biological conditions. As this is a relatively new delivery technology, it has currently only been utilized in an *in vitro* setting. In 2015, the group repurposed the nanoclew for CRISPR/Cas9 delivery by designing the palindromic sequences to be partially complementary to the sgRNA within the Cas9:sgRNA ribonucleoprotein complex (Figure 8(A-a)) (Sun et al., 2015). By coating the nanoclew with PEI to induce endosomal escape (Figure 8(A-b)), the group demonstrated roughly 36% efficiency in delivery of CRISPR/Cas9 RNP with the nanoclew (compared with 5% with bare Cas9:sgRNA and PEI). This allowed the nanoclew to attain efficiencies on the order of other high-efficiency CRISPR/Cas9 delivery systems, but still contain no viral components (or indeed, any exogenous material besides repeating DNA and PEI). More testing is warranted, particularly on the potential immunogenicity of DNA nanoclews. Still, early results are promising for this new delivery system.

### Gold nanoparticles (AuNPs)

AuNPs have many uses in applied biomedical science, from imaging agents to inert carriers of other components. As such, these particles are readily used in *in vitro*, *ex vivo*, and *in vivo* settings. Mout et al. (2017) demonstrated that, by engineering Cas9:sgRNA RNP and AuNPs to associate with one another (Figure 8(B)), a complex is created that can be efficiently delivered to cells and cause a desired mutation at a rate of roughly 30%. Lee et al. (2017) also reported use of AuNPs to deliver Cas9:sgRNA RNP to mice suffering from DMD. In this work, 15 nm diameter AuNPs were conjugated to thiolated short DNA oligos (Figure 8(C)), which were then conjugated to a single-stranded donor DNA. This donor DNA then complexed with the Cas9 RNP. The resulting particle was coated in a silicate and an endosomal disruptive polymer, PAsp(DET). A single injection of the AuNP-Cas9 conjugate corrected 5.4% of the mutated DMD-causing dystrophin gene and showed recovered dystrophin gene expression. Treated mice further showed partial restoration of muscle function and reduced levels of fibrosis.

Again, these results place AuNPs within the high bounds for CRISPR/Cas9 delivery efficiency while also eliminating the need for exogenous viral material. In addition, unlike the DNA nanoclew which relies on a biological molecule to act as a carrier, AuNPs are inert and will not trigger an immune response to the nanoparticle itself (Lee et al., 2017). Still, AuNPs have been shown to stimulate immune cytokine production in general (for a recent review, see Dykman and Khlebtsov, 2017). While this method also requires additional testing, it is promising as another delivery mechanism for CRISPR/Cas9 components.

### iTOP

Many other delivery techniques have been developed for gene editing systems. An example includes the CPP-independent protein delivery method reported by D'Astolfo et al. (2015). This technique, termed iTOP for induced transduction by osmocytosis and propanebetaine, uses NaCl-mediated hyperosmolality together with a transduction compound (propanebetaine) to trigger macropinocytotic uptake into cells of extracellular macromolecules. As another newer delivery technique, applications of iTOP have been limited to *in vitro* settings at this time. iTOP was used to delivery CRISPR/Cas9 RNP into primary human KBM7 and H1 cells, conferring diphtheria toxin resistance with ∼70% gene knockout efficiency.

## Emerging delivery technologies

We conclude this review with a look at four intriguing technologies: streptolysin O (SLO), multifunctional envelope-type nanodevices (MENDs), lipid-coated mesoporous silica particles, and inorganic nanoparticles. While none of these have been demonstrated in the literature for CRISPR/Cas9 delivery, their properties make them naturally amenable for use as CRISPR/Cas delivery vehicles.

### SLO

SLO is a toxin produced by Group A streptococci that works by creating pores in mammalian cell membranes (Sierig et al., 2003). While typically fatal to cells, Walev et al. (2001) developed a system in 2001 to allow for this toxin to act in a reversible manner. This allows for the delivery of proteins of up to 100 kDa to the cytosol of both adherent and non-adherent cells in culture without compromising overall viability. Other groups have used SLO for delivery of siRNA (Brito et al., 2008) and imaging agents for live-cell microscopy (Teng et al., 2017). Although there would be clear challenges to using SLO *in vivo*, the potential is there for its usage *in vitro* for delivery of CRISPR/Cas9 components, primarily for the smaller variants of Cas9.

### MENDs

MENDs, developed by the Harashima group at Hokkaido University, are a non-viral gene editing and therapeutic delivery system that is composed of condensed plasmid DNA, a PLL core, and a lipid film shell (Kogure et al., 2004). Packaging the DNA/PLL core with lipids increased transfection rates by ten-fold over bare DNA/PLL. Addition of the cell-penetrating peptide stearyl octaarginine to the lipid shell increased transfection rates by 1000-fold over bare DNA/PLL. The lipid envelope can be readily modified with other functional components, including the following: polyethylene glycol to increase vascular circulation time, ligands for targeting of specific tissues/cells, additional cell-penetrating peptides for greater cellular delivery, lipids to enhance endosomal escape, and nuclear delivery tags.

Recently, a tetra-lamellar MEND (T-MEND) was developed that targeted the cellular nucleus and mitochondria, and a PEG-peptide-DOPE-conjugated MEND (PPD-MEND) was developed that targeted bladder cancer cells (Nakamura et al., 2012). MEND has been used to successfully deliver cargoes of plasmid DNA, short interfering RNA (siRNA), and Bacillus Calmette-Guerin (BCG) cell wall therapeutic agents. This versatile platform may serve as an effective CRISPR/Cas9 delivery tool in the future, although as with SLO, more work must be done to move the work from *in vitro* to *in vivo* settings.

### Lipid-coated mesoporous silica particles

Developed by Brinker and colleagues at Sandia National Laboratories and the University of New Mexico, this biological delivery system is composed of a mesoporous silica nanoparticle core and a lipid membrane shell (Liu et al., 2009). While not yet utilized for CRISPR/Cas9, the particles have intriguing properties that may make them good delivery vehicles for the technology. The silica core has a large internal surface area, leading to high cargo loading capacities. In addition, pore size, pore chemistry, and overall particle size can be individually tailored, allowing for the loading of various types of cargo (Du et al., 2014; Durfee et al., 2016). The lipid coating of the particle can also be tailored to maximize cargo loading, increase circulation times, and provide precise targeting and cargo release. A wide variety of lipids and lipid modifications have been used in the formulation of lipid-coated mesoporous silica particles, allowing selection of the most relevant lipid formulation for the selected cargo and application (Liu et al., 2009; Mackowiak et al., 2013; Wang et al., 2013; Du et al., 2014; Durfee et al., 2016; Gonzalez Porras et al., 2016; Su et al., 2017).

To date, lipid-coated mesoporous silica particles have been loaded with a variety of imaging agents, chemotherapeutics, and phototherapy agents for both *in vitro* and *in vivo* work (Mackowiak et al., 2013; Wang et al., 2013; Durfee et al., 2016; Su et al., 2017). The characteristics of this delivery platform seem to naturally lend themselves to CRISPR/Cas9 components. Still, there are many challenges to overcome, primarily the packaging of large cargoes. CRISPR/Cas9 components, whether in an RNP complex, as mRNA, or as DNA plasmids, are larger than other components that have been reported to load within the particles.

### Inorganic nanoparticles

Inorganic nanoparticles are natural potential CRISPR component carriers because they have already been used for similar purposes. Examples of these include AuNPs, carbon nanotubes (CNTs), bare mesoporous silica nanoparticles (MSNPs), and dense silica nanoparticles (SiNPs). The use of AuNPs for CRISPR/Cas9 delivery was described above. While CNTs (Bates and Kostarelos, 2013), MSNPs (Luo et al., 2014), and SiNPs (Luo and Saltzman, 2000) have been used for many gene delivery applications, the use of these carries for Cas9 delivery has yet to be reported. However, when compared with viral and lipid/polymer based vectors, inorganic nanoparticles are simpler to generate, with reproducible composition, size, and size distribution, are simpler to characterize and chemically functionalize, and are more stable over time. We, therefore, expect many reports detailing the use of inorganic nanoparticles as delivery vehicles for CRISPR/Cas9 in both *in vitro* and *in vivo* settings in the short term.

## Perspective and future directions

There are many benefits of CRISPR/Cas9 systems that can be utilized using *in vitro* laboratory engineering. However, full realization of the potential of CRISPR/Cas9 approaches will require addressing many challenges. Within the system itself, off-target cutting remains a problem. Cas9 nickases and mutants that reduce non-specific DNA binding have been engineered specifically to ameliorate this problem, though they are an imperfect solution. Extensive efforts have been made in understanding sgRNA binding and mismatch tolerance, leading to the development of several predictive software sgRNA design tools; however, our understanding of off-target effects remains poor. This is a vital area for continued study if CRISPR/Cas9 is to realize its promise.

Regarding gene cargo delivery systems, this remains the greatest obstacle for CRISPR/Cas9 use, and an all-purpose delivery method has yet to emerge. Instead, multiple methods are seen for delivering CRISPR to cells. Every method has both advantages and disadvantages, and some can be quite specific or ill-suited to certain types of delivery (e.g. delivery to cells in a flask vs. delivery to a living organism). Further, the best gene editing results with minimal off-target effects are generally obtained from delivery of the ribonucleoprotein, as opposed to plasmid DNA or mRNA. Currently, there are more options for delivery of small-molecule cargo than for the relatively large protein–nucleic acid complex. Development of new delivery approaches that enable effective RNP delivery will make a meaningful impact to the field. Still another barrier for delivery systems is ensuring that the chosen system is both safe and specific. Safety in living organisms will always be a concern, and a delivery vehicle that can target the desired cells with high-specificity will also limit off-target effects and improve safety. Additionally, it is vitally important that, especially in the case of nanoparticle carriers, long-term studies on safety of the component pieces are done. There is currently limited information available on where various components of nanoparticle delivery systems end up in the body, how long they stay there, and whether there is any long-term toxicity associated with any component.

As evident through the many examples presented in the ‘Delivery Methods’ portion of this review, the therapeutic potential of CRISPR/Cas9 is great. Already, much has been published on the altering of cell line genotypes and phenotypes using this gene editing system. Work has even moved into animal models, and therapeutic effects are broad-ranging, including inhibition of viral infection, reversal of debilitating conditions such as muscular dystrophy, and elimination of tumors in cancer models. Taken together, it is easy to see the reason for so much excitement in the CRISPR field. As the technology evolves and CRISPR becomes even more mechanistically precise and can be delivered with ever-increasing precision, its therapeutic potential will continue to rise.

Importantly, the CRISPR field is evolving at an incredible pace, with the number of peer-reviewed scientific papers with the term CRISPR in the title or abstract increasing by 1,453% since 2011. The outlook for the technology, therefore, is certainly positive, and we expect that with the large number of researchers from divergent fields now focusing on this system, any limitations will eventually be addressed and solved. Indeed, CRISPR is even beginning to make its way into modern-day popular culture, with casual references in multiple media formats. Truly, CRISPR is the new face of modern genetic engineering.
